# Hugh McGinnis Grant

**Published:** 1888-01

**Authors:** 


### Obituary.

---

HUGH MCGINNIS GRANT, D. D. S.—One of the most widely known dental practitioners in the Southwest died in Abingdon, Virginia, where he had been engaged in practice for many years, December 19th, 1887.

Dr. Grant was a class-mate and the room-mate of the editor of this JOURNAL, having graduated in the Baltimore College of Dental Surgery in the class of 1855.

At different times he held positions in the faculties of several dental colleges; as Demonstrator of Operative Dentistry for one term in the Baltimore College of Dental Surgery and Professor of Clinical Dentistry in the former New Orleans Dental College. Dr. Grant was also at different times President of the Southern Dental and of the Virginia State Dental Associations, and was a gentleman of positive traits and ideas, and very enthusiastic for the advancement of his profession.

As a student and practitioner, Dr. Grant was earnest, diligent and persevering, and stood well in his profession, being a good citizen and a kind husband and father. His death will be a sad bereavement to many friends, and be regretted by all who knew him.

Very few of the class in which he graduated survive him, and his presence will be sadly missed at the meeting of the Southern and State Dental Associations, in both of which he was a leading member.

At a meeting of the members of the dental profession of Abingdon, the following preamble and resolutions were adopted:

We, with profound sorrow and regret, learn of the death of Dr. H. M. Grant, whom, it has pleased God to remove from the world, at the zenith of his profession, and, with the highest esteem of the citizens of this community, where he has so long resided. To few men is it allotted to attain to such excellence in their chosen profession, and few have done so much to advance the interests of dentistry.

Though Dr. Grant was incessantly occupied with the cares of a large practice, yet he always found time to lend a helping hand to the Associations of the profession, seeking to guide them by the light of his experience, in their struggle along the road over which he had so successfully passed. Therefore be it

*Resolved*, That in the death of Dr. Grant, Abingdon has lost one of her valued citizens, and the dentists an illustrious exampler and a beloved co-laborer.

*Resolved*, That we heartily tender to the bereaved family our sincere sympathy, commending them to Him whose ways are past finding out—too wise to err, too good to be unkind.

*Resolved*, That a copy of these resolutions be sent to the family, and published in the Abingdon papers.

R. T. McQuown,<br>

J. H. Morgan.

December 20th, 1887.

